# Magnetically Separable Fe_3_O_4_/AgBr Hybrid Materials: Highly Efficient Photocatalytic Activity and Good Stability

**DOI:** 10.1186/s11671-015-0952-x

**Published:** 2015-06-03

**Authors:** Yuhui Cao, Chen Li, Junli Li, Qiuye Li, Jianjun Yang

**Affiliations:** Key Laboratory for Special Functional Materials of Ministry of Education, Henan University, Kaifeng, 475004 China; Collaborative Innovation Center of Nano Functional Materials and Applications, Henan Province, China

**Keywords:** AgBr, Fe_3_O_4_, Magnetic separation, Visible light, Photocatalysis

## Abstract

Magnetically separable Fe_3_O_4_/AgBr hybrid materials with highly efficient photocatalytic activity were prepared by the precipitation method. All of them exhibited much higher photocatalytic activity than the pure AgBr in photodegradation of methyl orange (MO) under visible light irradiation. When the loading amount of Fe_3_O_4_ was 0.5 %, the hybrid materials displayed the highest photocatalytic activity, and the degradation yield of MO reached 85 % within 12 min. Silver halide often suffers serious photo-corrosion, while the stability of the Fe_3_O_4_/AgBr hybrid materials improved apparently than the pure AgBr. Furthermore, depositing Fe_3_O_4_ onto the surface of AgBr could facilitate the electron transfer and thereby leading to the elevated photocatalytic activity. The morphology, phase structure, and optical properties of the composites were characterized by scanning electron microscopy (SEM), X-ray diffraction (XRD), UV–visible diffuse reflectance spectra (UV–vis DRS), and photoluminescence (PL) techniques.

## Background

Up to now, most of the silver oxide and silver halide have attracted much attention because of their strong visible light absorption performance [1–7]. Particularly, AgBr, which has a band gap of 2.6 eV, is well known as a photosensitive material and has been extensively applied to photographic films, which demonstrated excellent performance in degradation of dye pollutants and decomposition of water [8–10]. For example, Ag/AgBr/TiO_2_ [11], Ag–AgBr/TiO_2_/RGO [12], AgBr(I)@Ag [13], Fe(III)/AgBr [14], and Ag/AgBr/ZnO [15] have been successfully fabricated by diverse techniques, and their novel and unique photocatalytic properties have been extensively explored.

For the nanosized or microsized photocatalysts, effective separation from the mixed system and recycle using are important problems to restrain their real applications [16, 17]. Immobilizing catalysts on magnetic substrates by feasible methods is proven to be an effective approach for removing and recycling particles [18–21]. Moreover, Fe_3_O_4_ has excellent conductivity, so it could act as an electron transfer channel and acceptor, which could suppress the photo-generated carrier recombination. For instance, Ye et al. reported that the hierarchical core–shell-structured Fe_3_O_4_/WO_3_ has a more effective photoconversion capability than pure WO_3_ or Fe_3_O_4_ [22]. The Ag halides such as AgBr and AgI are photoactive to visible light. When they were immobilized on SiO_2_@Fe_3_O_4_ magnetic supports, they exhibited faster degradation rates for 4-chlorophenol than N-TiO_2_ [23]. However, the Ag halides were easily photoreduced and losed their stability quickly.

The motivation of the present research originated from the idea that Fe_3_O_4_ has high conductivity and its CB level (1 V vs. NHE) makes it become a good candidate for coupling with AgBr. Based on the above reason, we prospect their combination could improve the photocatalytic performance by enhancing charge transport. Herein, conductive Fe_3_O_4_ particles and visible light active AgBr were coupled together to prepare the magnetically recyclable Fe_3_O_4_/AgBr composites with visible light activity. Studies of their photocatalytic performance in the decomposition of methyl orange (MO) indicated that Fe_3_O_4_/AgBr photocatalysts exhibited excellent catalytic activity under visible light illumination. Meanwhile, the stability of AgBr was improved when it was coupled with Fe_3_O_4_.

## Methods

### Preparation of the Photocatalyst

#### Synthesis of Fe_3_O_4_ Nanospheres

The Fe_3_O_4_ nanospheres were prepared according to the literature reported previously [24]. In a typical synthesis, 0.5 g of 1 g FeCl_3_ · 3H_2_O, 3.0 g NaAc, and 10 mL oleic acid were added to 30 mL ethylene glycol into a three-necked flask, and then a red solution was formed. The mixture was stirred vigorously at 50 °C for 20 min until all reagents were dissolved completely. Then, the mixture was transferred into a Teflon-lined autoclave and heated at 200 °C for 20 h. The products were cooled down to room temperature, washed with ethanol for several times, and dried under vacuum to give a black solid.

#### Synthesis of Fe_3_O_4_/AgBr Hybrid Materials

Fe_3_O_4_ nanospheres (0.01 g) were dispersed in 20 mL deionized water and then ultrasonically dispersed evenly. AgNO_3_ (1.18 g) was added into the solution, and then NaBr (0.1 mol/L) was added dropwise slowly. The resulting suspensions were filtered, washed several times with distilled water, and finally dried in vacuum. Different Fe_3_O_4_/AgBr samples were obtained by adjusting the mass ratio of Fe_3_O_4_ and AgBr, and the sample was denoted as Fe_3_O_4_/AgBr-*x* (*x* means the percentage of Fe_3_O_4_).

### Characterization

X-ray diffraction (XRD) patterns were measured on an X’Pert Philips diffractometer (Cu Kα radiation, 2*θ* range 10°–90°, step size 0.08°, accelerating voltage 40 kV, applied current 40 mA). The morphology of the samples was taken on a Hitachi S-4800 scanning electron microscope (SEM). UV–visible diffuse reflectance spectra (UV–vis DRS) were obtained on a Shimadzu U-3010 spectrometer, using BaSO_4_ as a reference. The photoluminescence (PL) spectra were recorded on a F-7000 FL spectrophotometer.

### Evaluation of the Photocatalytic Activity

MO was selected as the model pollutant to evaluate the photocatalytic activity of the Fe_3_O_4_/AgBr hybrid materials. In a typical experiment, 0.1 g of the photocatalyst was put into a 120 mL quartz reactor containing 100 mL MO aqueous suspension (20 mg/L, pH = 7). Prior to irradiation, the suspension was magnetically stirred in the dark for 30 min to establish an adsorption–desorption equilibrium. A 300-W Xe arc lamp with a 420 cutoff filter was used as the light source (*λ* ≥ 420 nm, *I*_420_ = 8.0 mW/cm^2^). At 2-min intervals, 5 mL of the suspension was collected and centrifuged for 3 min to remove the catalyst particulates for analysis. The residual MO concentration was detected at 464 nm using a UV–vis spectrophotometer (722, Shanghai Jingke Instrument Plant, China).

## Results and Discussion

### Phase Structure and Morphology of the Samples

**Fig. 1:**
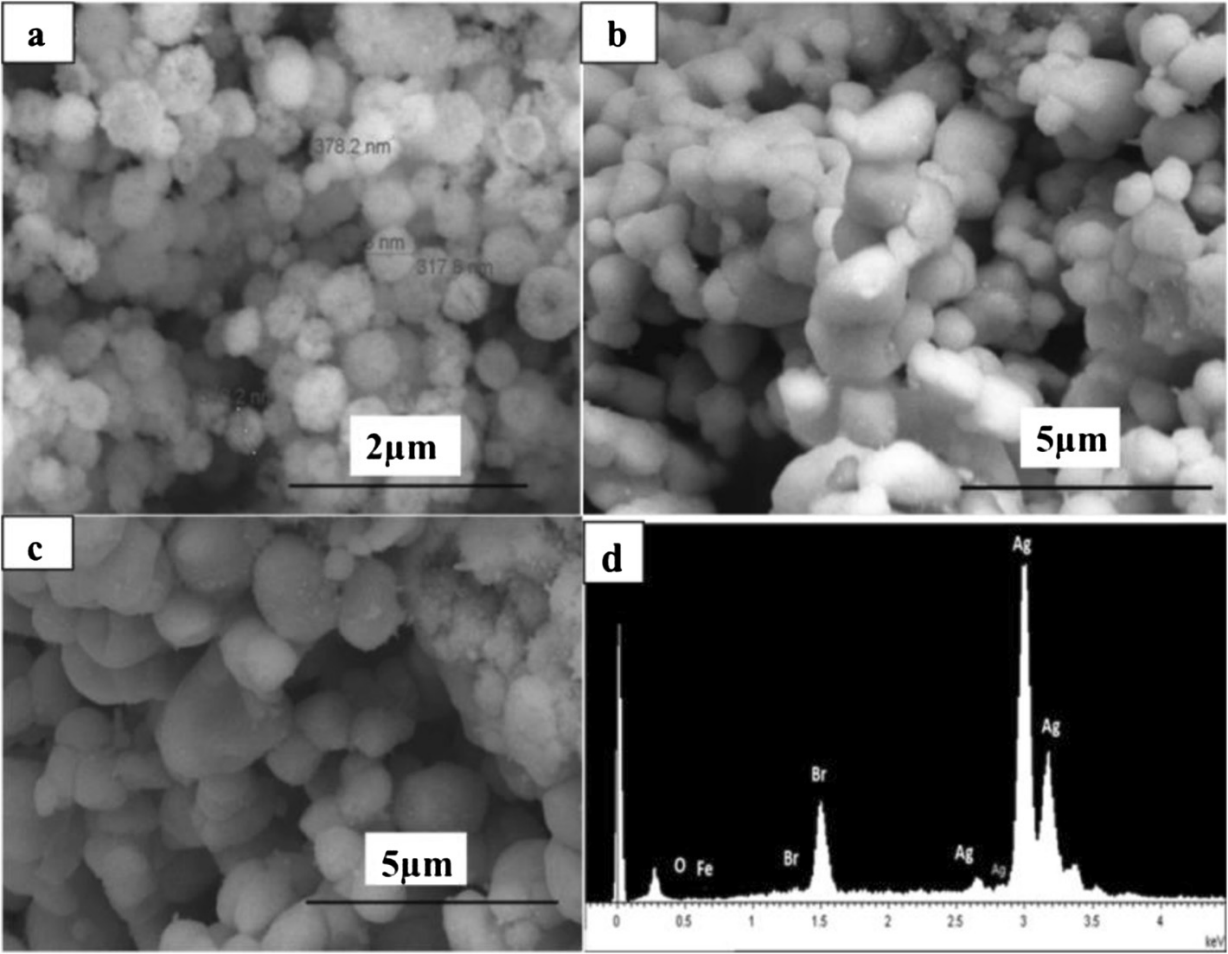
SEM images of the Fe_3_O_4_/AgBr hybrid materials obtained. **a** Fe_3_O_4_. **b** AgBr. **c** Fe_3_O_4_/AgBr-0.5. **d** EDS images of Fe_3_O_4_/AgBr-0.5

Figure 1a shows that the size of Fe_3_O_4_ nanospheres was about 100 ~ 200 nm. The surface of Fe_3_O_4_ particles was rough, and each magnetic microsphere was constructed with many small magnetic grains. From Fig. 1b, we can clearly see that the obtained AgBr particles by the precipitation method easily agglomerate to large particles and their size was more than 300 nm. Figure 1c displays that when Fe_3_O_4_ was coupled with AgBr, the particle size of the composite increased apparently than the pure AgBr particles. The magnetic property of the surface Fe_3_O_4_ would result in the agglomeration of the particles. The EDS spectrum of Fe_3_O_4_/AgBr-0.5 hybrid materials indicates that the atomic ratio of Fe and Ag is approximately 1:134, which is a little larger than the designed value.

**Fig. 2:**
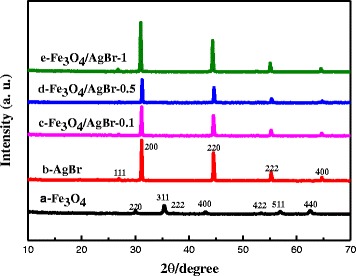
XRD pattern of the photocatalysts obtained. (**a**) Fe_3_O_4_. (**b**) AgBr. (**c**) Fe_3_O_4_/AgBr-0.1. (**d**) Fe_3_O_4_/AgBr-0.5. (**e**) Fe_3_O_4_/AgBr-1

Figure 2 shows the typical XRD patterns of the as-prepared Fe_3_O_4_/AgBr hybrid materials with different Fe_3_O_4_ contents, which matched well with those of Fe_3_O_4_ (magnetite, JCPDS 85-1436) [22]. The diffraction peaks of pure AgBr at 26.8°, 30.9°, 44.3°, 55.0°, and 64.5° were assigned to the (111), (200), (220), (222), and (400) crystal planes of AgBr (JCPDS 06-4308) [14]. With increasing Fe_3_O_4_ content, no characteristic peaks were ascribed to Fe_3_O_4_ emerging with AgBr phase, which should be due to the lower content of Fe_3_O_4_.

### Optical Properties of the Photocatalysts

**Fig. 3:**
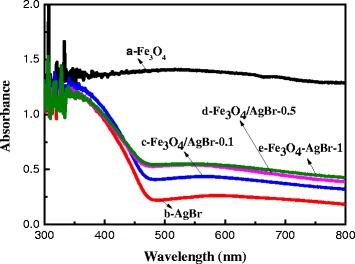
UV–vis diffuse reflectance spectra (DRS) of Fe_3_O_4_/AgBr hybrid materials obtained. (**a**) Fe_3_O_4_. (**b**) AgBr. (**c**) Fe_3_O_4_/AgBr-0.1. (**d**) Fe_3_O_4_/AgBr-0.5. (**e**) Fe_3_O_4_/AgBr-1

The UV–vis spectra of Fe_3_O_4_/AgBr hybrid materials are illustrated in Fig. 3. The pure Fe_3_O_4_ particles show strong absorption both in ultraviolet and visible light regions, which may be attributed to its small band gap. The absorption band edge of AgBr was about 470 nm, so the calculated band gap was 2.64 eV. AgBr was often used as a good visible light sensitizer because it exhibited a strong absorption in the visible light. After loading Fe_3_O_4_ on AgBr particles, the visible light absorption increased significantly. And as the increase with the loading content of Fe_3_O_4_, the visible absorption of the composites enhanced gradually, indicating that the existence of Fe_3_O_4_ could promote visible light absorption effectively.

### Photocatalytic Activity for MO Degradation on Fe_3_O_4_/AgBr Hybrid Materials

**Fig. 4:**
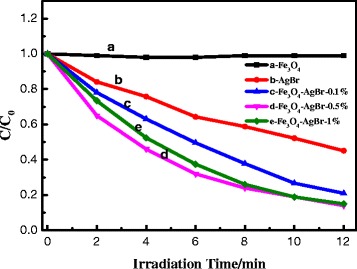
Visible light responded photodegradation of MO on the series of photocatalysts obtained. (**a**) Fe_3_O_4_. (**b**) AgBr. (**c**) Fe_3_O_4_/AgBr-0.1. (**d**) Fe_3_O_4_/AgBr-0.5. (**e**) Fe_3_O_4_/AgBr-1

**Fig. 5:**
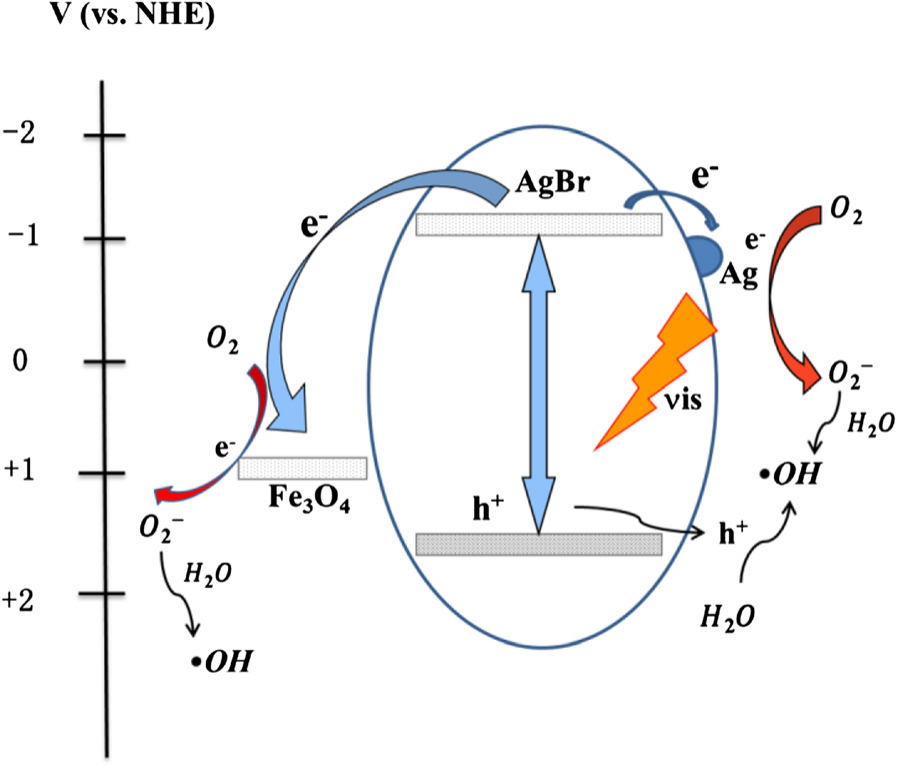
Photocatalytic mechanism of MO degradation on Fe_3_O_4_/AgBr hybrid materials under visible light illumination

**Fig. 6:**
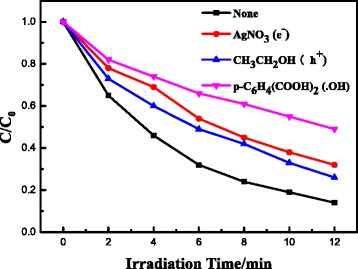
Detection of the active species of electrons, holes, and hydroxyl radicals

The photocatalytic performances of the photocatalysts were evaluated by photoinduced decolorization of MO aqueous solution, as shown in Fig. 4. Prior to irradiation, the mixed solution of MO and photocatalyst was kept in the dark for 30 min to obtain an adsorption/desorption equilibrium. For comparison, the photocatalytic activity of the pure AgBr was tested and the degradation yield reached approximately 55 % in 12 min. When Fe_3_O_4_ nanospheres were loaded on AgBr particles, the photocatalytic activity increased apparently than the pure AgBr. The photocatalytic mechanism of Fe_3_O_4_/AgBr composites for MO degradation under visible light is illustrated in Fig. 5. The CB level of Fe_3_O_4_ (1 V vs. NHE) is much lower than that of AgBr (−1.1 V vs. NHE) [22–25], so the photo-excited electrons on the conduction band (CB) of AgBr can transfer to the CB of Fe_3_O_4_. And the conductivity of Fe_3_O_4_ is as high as 1.9 × 10^6^ S m^−1^; the electrons on Fe_3_O_4_ particles would transfer out quickly and react with the surface pollutants. Meanwhile, Ag nanoparticles on the surface of AgBr can act as electron capture traps to improve the separation efficiency of the charge carriers and thereby improving the photocatalytic efficiency. These should be the main reason for the enhancement of the photocatalytic activity for Fe_3_O_4_/AgBr composites. In addition, the loading amount of Fe_3_O_4_ particles has an effect on the activity of the composites. The sample Fe_3_O_4_/AgBr-0.5 has the best photocatalytic activity; the degradation yield of MO reached nearly 85 % within 12 min. In order to clarify the reasons for this result, the active species in photodegradation process of MO were detected. Methanol, silver nitrate, and terephthalic acid solution were added into MO dye solution to capture electrons, holes, and · OH, respectively. As can be seen from Fig. 6, when the active species of electrons, holes, and · OH were captured, the degradation yield of MO decreased from 85 % to 68 %, 74 %, and 51 %, respectively. That indicated · OH and electrons played more important roles comparing the holes in the photodegradation of MO.

**Fig. 7:**
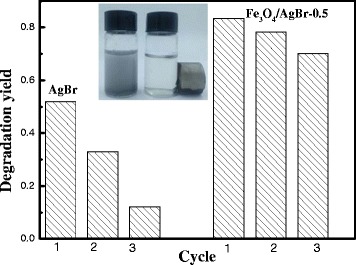
The stability of the pure AgBr and Fe_3_O_4_/AgBr-0.5 hybrid materials on MO photodegradation in the consecutive three cycles. The inset shows that Fe_3_O_4_/AgBr-0.5 composites have a certain magnetic response to an applied magnetic field

As well known, AgBr is not stable, and it often suffers photo-corrosion. So, the stability of AgBr and Fe_3_O_4_/AgBr-0.5 was evaluated. As shown in Fig. 7, the photocatalytic activity on the pure AgBr decreased sharply in the consecutive three cycles. The degradation yield of MO on the pure AgBr particles in the tree cycles was 0.52, 0.33, and 0.12, respectively. The photo-excited electrons on AgBr would reduce Ag^+^ to the metallic Ag, and the small Ag nanoparticles would cover on the surface of AgBr. And the surface Ag nanoparticles would prohibit the photo-absorption of the inner AgBr. When the amount of Ag was enough, the photo-excitation of the inner AgBr would be hold back, and as a result, the photocatalytic activity decreased remarkably as the reaction proceeding. However, for the Fe_3_O_4_/AgBr hybrid materials, the stability was much better. The degradation yield of MO on Fe_3_O_4_/AgBr composites was 0.83, 0.78, and 0.71 in the consecutive cycles, respectively. The Fe_3_O_4_ particles on the surface could transfer the photo-excited electrons out quickly, which inhibit the self-reduction of AgBr. So, the long-term stability of Fe_3_O_4_/AgBr composites was obtained than the pure AgBr. In the photocatalytic application, effective recycling of catalyst is very important. Thanks to the existence of Fe_3_O_4_, the catalyst has magnetism, which is favorable for recycling. As shown in the inset of Fig. 7, Fe_3_O_4_/AgBr-0.5 composites could be easily separated from the suspension by an external magnetic field. As expected, the as-prepared Fe_3_O_4_/AgBr composites exhibited a certain magnetic response.

### Photoluminescence of the Series of Photocatalysts

**Fig. 8:**
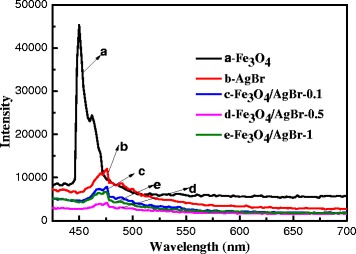
Photoluminescence (PL) spectra obtained. (**a**) Fe_3_O_4_. (**b**) AgBr. (**c**) Fe_3_O_4_/AgBr-0.1. (**d**) Fe_3_O_4_/AgBr-0.5. (**e**) Fe_3_O_4_/AgBr-1

The fluorescence spectrum can provide much more information about carrier capture, migration, conversion, separation, etc., so it has been used for measuring the separation of the photo-generated electron–hole pairs [26]. The emission signals in the fluorescence spectrum are mainly from the recombination of the photo-generated electron–hole pairs, and the lower fluorescence intensity often implies the higher separation efficiency of the charge carriers. Figure 8 shows the fluorescence spectra of the samples in a wavelength range of 400–700 nm. It can be seen that the peaks were similar except Fe_3_O_4_. No characteristic peaks were ascribed to Fe_3_O_4_ emerging with Fe_3_O_4_/AgBr composites, which should be due to the lower content of Fe_3_O_4_. Moreover, Fig. 8 also shows a decrease in emission intensity from AgBr to Fe_3_O_4_/AgBr samples, indicating that an appropriate amount of Fe_3_O_4_ could significantly reduce the recombination rate of photo-generated electrons and holes of AgBr. The PL intensity of the Fe_3_O_4_/AgBr-0.5 sample was the lowest, which indicated that the separation efficiency of charge carriers was the highest. That was in accord with the photocatalytic activity result very well.

## Conclusions

Fe_3_O_4_/AgBr hybrid materials with high photocatalytic efficiency under visible light were prepared through the precipitation method. The Fe_3_O_4_/AgBr samples showed much higher photocatalytic activity than the pure AgBr, which was due to the matched band structure of two components and the higher conductivity of Fe_3_O_4_. When the loading amount of Fe_3_O_4_ was 0.5 %, the highest photoactivity was obtained, and the degradation yield of MO reached 85 % within 12 min. The PL spectra indicated that Fe_3_O_4_/AgBr hybrid materials had the higher separation efficiency of the photo-excited charge carriers, and that was in accordance with the photocatalytic activity very well. In addition, the stability of Fe_3_O_4_/AgBr composites was improved comparing with the pure AgBr. The photo-excited electrons would transfer out quickly from the surface Fe_3_O_4_, so the self-reduction of AgBr to metallic Ag was prohibited, and as a result, the long-term stability of Fe_3_O_4_/AgBr was obtained.
